# ASO Author Reflections: Reflections on the Impact of Access to CRS/HIPEC via Medicaid Expansion as a Key to Future Models for Advanced Surgical Cancer Care

**DOI:** 10.1245/s10434-024-16556-3

**Published:** 2025-01-05

**Authors:** David Caba Molina

**Affiliations:** 1 Department of Surgery, Division of Surgical Oncology, Loma Linda University, Loma Linda, USA; 2 Department of General Surgery, Riverside University Health System Medical Center, Moreno Valley, USA

After more than a decade of Medicaid expansion, the insurance-related benefits regarding outcomes in malignancy have been well-documented. Overall, Medicaid expansion has evened the playing field in access to specialized cancer treatments, with promising signs of narrowing survival disparities in the cancer-specific survival continuum. Access to affordable care and expanding the options for treatment in metastatic lower intestinal cancer was limited not only by insurance coverage but also by referral patterns and prevalence of specialized centers and surgeons offering cytoreductive surgery/hyperthermic intraperitoneal chemotherapy (CRS/HIPEC). This is likely due to improved utilization and acceptance of this treatment in the surgical oncology community. The impact of Medicaid expansion, early and late, is an interesting topic to study since reasonable comparisons can be made between states over the years using cancer-specific variables and parameters that affect survival in this population. Namely, travel distance provides compelling evidence supporting the hypothesis that improved access to healthcare in Medicaid expansion states may result in improved outcomes.

The sample size in our database analysis provides a stronger linear comparison and can capture changes over time within the same variables. This is critical when we consider the implementation of effective insurance coverage, which can take several years to show an impact of survival outcomes. In our manuscript, to be able to record the intervention of CRS/HIPEC, which is not present as a single procedure in the coding system of the National Cancer Database (NCDB), we used the timing of chemotherapy and surgery. This proved to be an effective tool to evaluate outcomes in this cohort when we combined appendiceal, colon, and rectal cancer as a single group. To our knowledge, this is the largest cohort incorporating the vast majority of patients in the United States for whom CRS/HIPEC was used.

Our study findings led to some interesting hypothesis-driving questions. We believe the disease-specific access to treatment in the current healthcare environment is related to referral pattern variables that are included in insurance coverage. These variables include (1) regional access to advanced cancer therapies, mainly surgical interventions for late-stage lower intestinal malignancies; (2) implementation of specialized treatments in local/regional centers rather than opting for centralized referrals to large medical centers; and (3) provider and patient education on alternative treatment options, including CRS/HIPEC, early in the disease diagnosis. These hypotheses are based on Medicaid being implemented in certain areas where there is a void in regional and local coverage. This will enable more direct referral patterns within a reasonable distance from diagnosis. In turn, this emphasizes the need for advanced cancer treatments such as HIPEC and CRS in regional centers that have undergone training in this therapeutic modality.

The public health benefits in complex cancer treatment and their relationship to financial burden should be studied in disease-specific sites and insurance coverage. This is an area currently being debated and analyzed when trying to promote equity in caring for patients with limited access to advanced cancer treatments such as CRS/HIPEC. Our data suggest that fragmented care and early referral plays an important role in cancer survival. We feel that improving access to advanced care should not be driven by insurance status or distance to higher level of care. The opportunity to treat advanced-stage lower gastrointestinal cancers begins with the emphasis on early referral and prompt approval. Medicaid expansion in our cohort showed improved outcomes and the implications should be broadened where advanced cancer is not viewed as a merely palliative disease, constrained by lack of insurance and limited access to care.^1–5^
